# Intramural Esophageal Hematoma With Associated Hemothorax

**DOI:** 10.14309/crj.0000000000000957

**Published:** 2023-01-13

**Authors:** Zilan X. Lin, Aaron Weiss, Bo Li, Shireen Pais

**Affiliations:** 1 Division of Gastroenterology and Hepatobiliary Diseases, Westchester Medical Center, Valhalla, NY; 2 Department of Medicine, Westchester Medical Center, Valhalla, NY

**Keywords:** esophageal hematoma, esophageal obstruction, hemoptysis, hemothorax

## Abstract

Intramural esophageal hematoma (IEH) is a rare manifestation of esophageal wall injury with nonspecific symptoms. It may be caused by trauma or occur spontaneously. IEH is often discovered on computed tomography or esophagogastroduodenoscopy and is typically managed conservatively with supportive care to allow healing. It is frequently an isolated finding in the esophagus and seldom involves any other organ. We report a rare case of a patient discovered to have an obstructing IEH with associated hemothorax after an unrelated surgical admission.

## INTRODUCTION

Intramural esophageal hematoma (IEH) occurs in rare instances of acute injury to the esophageal mucosa or submucosa. In contradistinction to Mallory-Weiss syndrome with mucosal tear and Boerhaave syndrome with full thickness perforation, IEH is characterized by an interlayer collection of blood in the esophageal wall. IEH can occur spontaneously or after trauma,^1,2^ with or without underlying coagulation disorders or the use of blood thinners. Typical presenting symptoms include dysphagia, odynophagia, chest pain, and/or hematemesis. Computed tomography (CT) is usually the initial imaging modality,^3^ followed by esophagogastroduodenoscopy (EGD) to establish the diagnosis.^1^ IEH generally is focal and follows a benign course.^4^ We report a rare case of a patient admitted for lower extremity ischemia, who was found to have an obstructing IEH and hemothorax.

## CASE REPORT

An 86-year-old woman with severe peripheral artery diseases (on apixaban at home) was admitted to the vascular surgery service for acute lower extremity ischemia. She was started on low-dose protocol heparin infusion at emergency department and taken to the operating room emergently, where she underwent thrombectomy with successful revascularization. No complication was noted by both surgical and anesthesia teams intraoperatively. The patient was transferred to the intensive care unit (ICU) postoperatively and continued on heparin infusion, 81 mg aspirin, and pantoprazole for stress ulcer prophylaxis. Of note, the patient had a known history of gastroesophageal reflux disease and had undergone an EGD 1 year prior at outside hospital and was found to have a hiatal hernia and Barrett's esophagus per verbal report.

**Figure 1.**
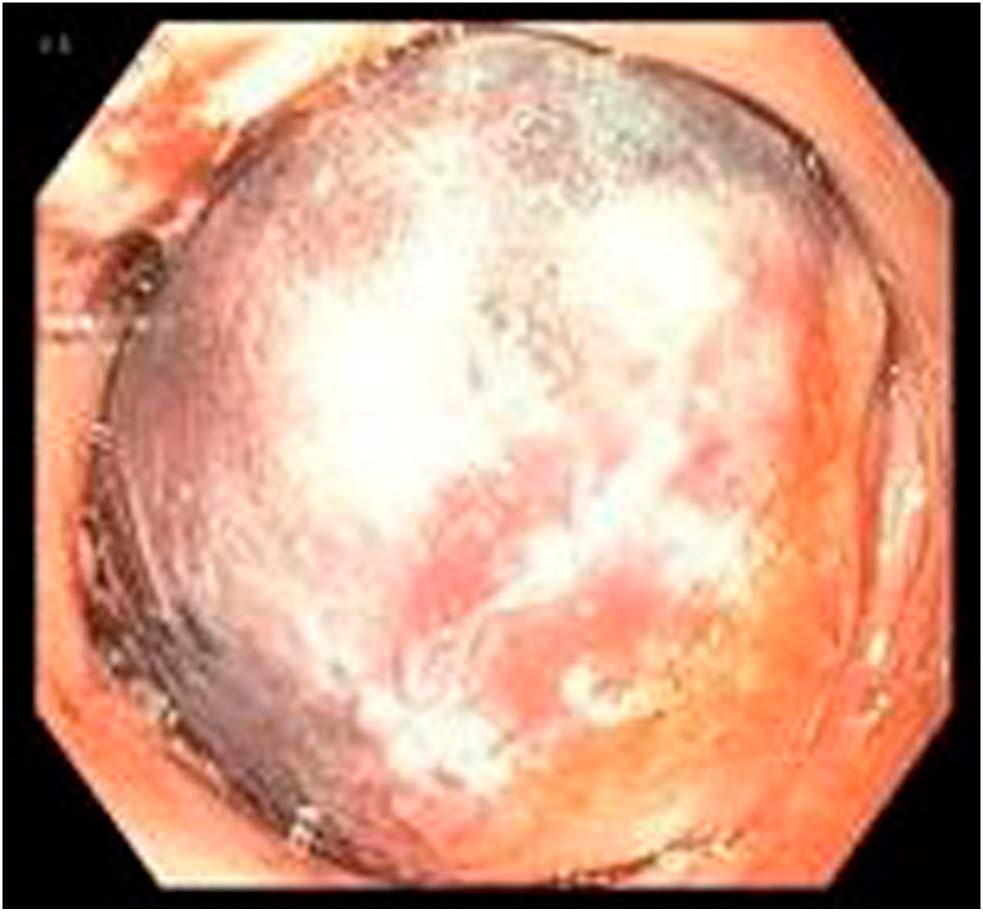
Endoscopic view of esophageal intramural hematoma obstructing the lumen.

**Figure 2.**
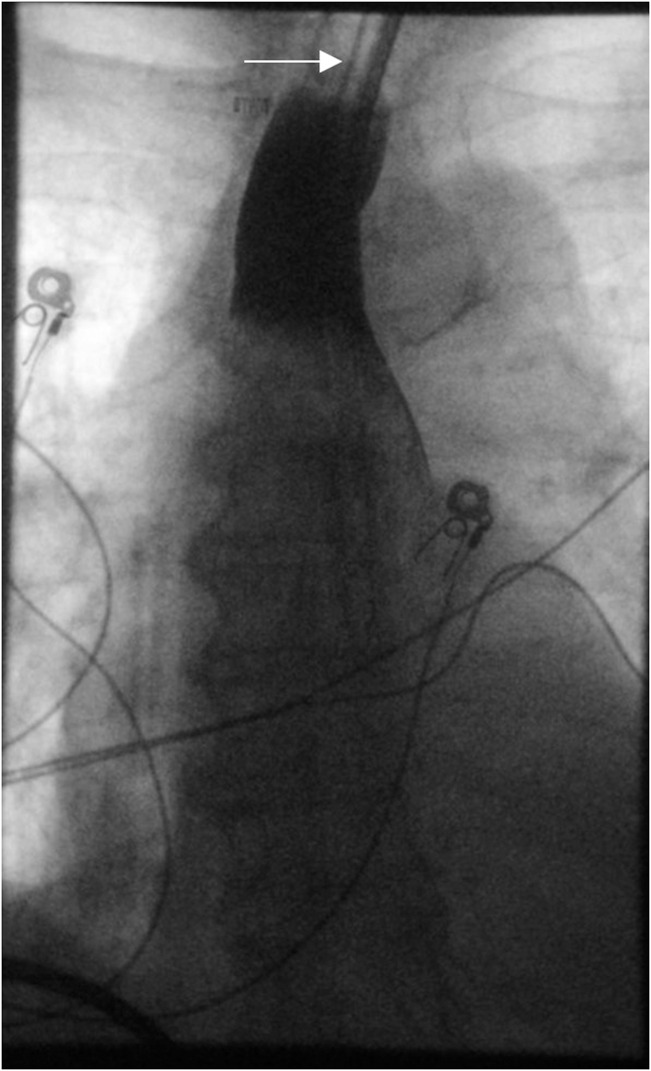
Esophagram showing contrast from nasogastric tube pooling in the proximal esophagus and a curvilinear margin (secondary to the mass effect). White arrow indicates the nasogastric tube.

**Figure 3.**
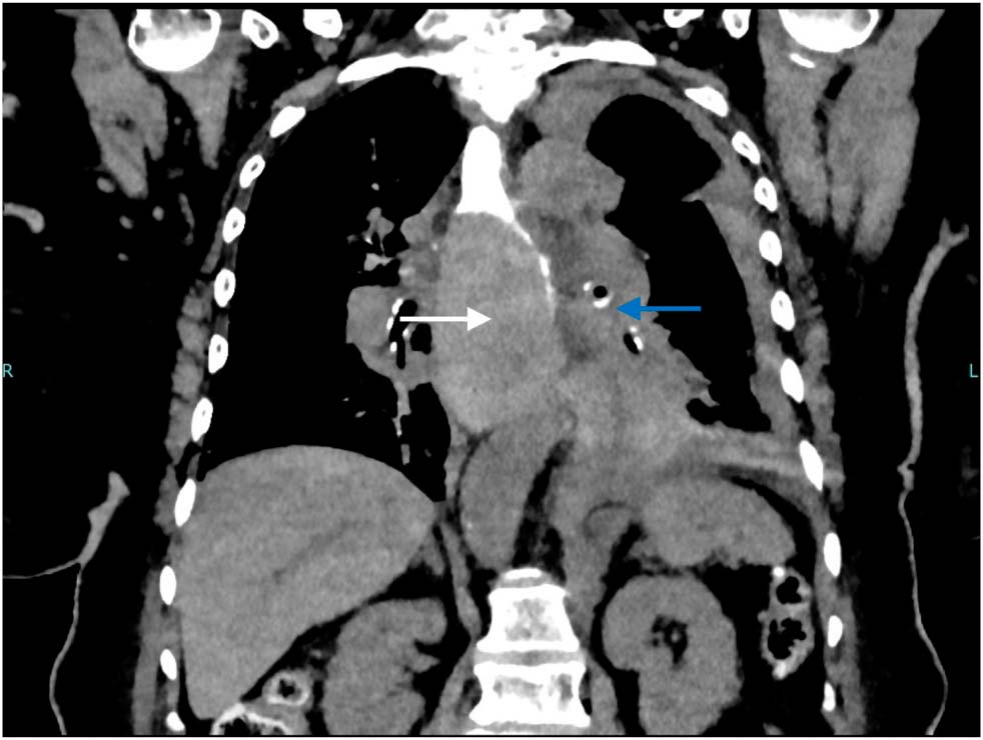
Contrast computed tomography scan showing esophageal hematoma (white arrow) with associated hemorrhagic effusion (blue arrow).

**Figure 4.**
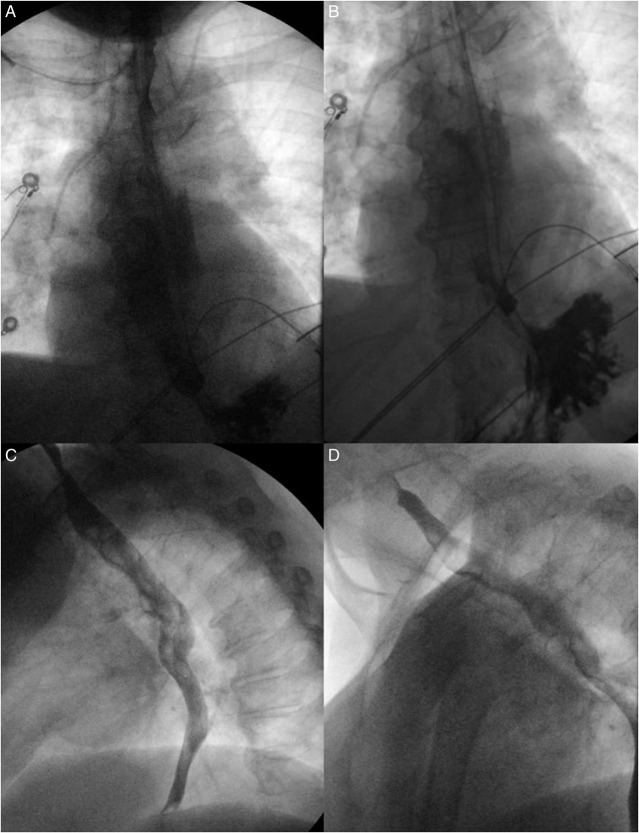
Esophagram showing transit of contrast into the stomach without extravasations on postoperative day 11 (A and B). Esophagram without evidence of contrast leak at 2 months postsurgery (C). Esophagram without evidence of contrast leak at 6 months postsurgery (D).

The patient's immediate perioperative course included several unsuccessful nasogastric tube (NGT) placements to advance into stomach by the ICU team for oral access but was otherwise uneventful. On postoperative day (POD) 1, she was noted to have epigastric pain, multiple episodes of vomiting with scant bloody emesis, and difficulty tolerating oral intake. A chest/abdomen/pelvic CT angiogram was obtained, and the results were negative for dissection or aneurysm but showed a small hiatal hernia with fluid in the esophagus to the level of the upper thorax. The patient was subsequently intubated without difficulty for airway protection. On POD 2, EGD was performed and revealed a dilated proximal esophagus with a large IEH occluding the esophagus from 24 to 30 cm from the incisors (Figure 1). The scope could not traverse the hematoma safely, so the decision was made to further evaluate the esophagus with noninvasive imaging. Meanwhile, a NGT was attempted to be placed by the ICU team at bedside. An esophagogram demonstrated the contrast material in the proximal to mid esophagus with a NGT in the proximal esophagus (Figure 2). A concomitant chest CT scan revealed interval increase in size of the IEH with blood tracking into the left crus, and a new left hemothorax (Figure 3). Given this finding, a chest tube was urgently placed and 350 mL grossly bloody fluid was immediately drained. In addition, the patient required blood transfusion because of hemoglobin drop from 15.2 g/dL at admission to 8.1 g/dL and temporary vasopressor support, while heparin infusion was discontinued. On POD 3, a repeat EGD again noted the occluding intramural hematoma, approximately 9 cm in size (28–37 cm from the incisors). A 14-French NGT was successfully advanced into the duodenum under endoscopic and fluoroscopic guidance and stayed in place thereafter. Pleural fluid culture grew multidrug resistant *Enterobacter cloacae*, which was treated with meropenem. The patient was managed conservatively with supportive care and was subsequently extubated and tube feeding was started. A repeat esophagogram on POD 11 showed transit of contrast material into the stomach, without any contrast extravasation (Figure 4). The patient continued to recover, tolerating a soft diet with resumption of anticoagulation at the time of her discharge to acute inpatient rehabilitation on POD 13. The outpatient follow-up esophagogram at months 2 and 6 demonstrated persistent healing (Figure 4).

## DISCUSSION

IEH may be caused by trauma, such as food impaction/foreign body ingestion,^5,6^ or iatrogenically after instrumentation. Although uncommon, IEH has been reported after NGT placement,^1^ esophageal variceal sclerotherapy,^7^ and cold forceps biopsy by EGD.^8^ It can also occur spontaneously after barotrauma or in the setting of coagulation disorders or the use of anticoagulation/antiplatelet therapy.^9–12^ Although not initially expected, our patient had a classic presentation with pain, inability to tolerate oral intake, and small volume hematemesis. The symptoms of IEH can mimic some devastating medical conditions such as aortic dissection, esophageal perforation, or malignancy, which must be ruled out.

IEH is an uncommon finding on upper endoscopy. Due to its rarity, no epidemiologic studies have been published, and only case reports are available in the current literature. EGD often confirms the diagnosis and rules out other more concerning illnesses. CT and contrast swallow studies are less invasive tools that can provide useful information to identify perforation and help guide diet advancement. Typically, IEH resolves spontaneously with supportive care and typically reveals an isolated esophageal finding, without involvement beyond the esophagus.^3,13^ Most patients with IEH can achieve full recovery with conservative treatment.

Our patient had an associated hemothorax, which while exceedingly rare, has been described. Guo et al^4^ reported on a patient with spontaneous IEH who developed clotted hemothorax requiring video-assisted thoracic surgery. Interestingly, in our case, the pleural fluid culture from the chest tube grew *E. cloacae*, a bacterium that may be acquired from the gastrointestinal tract. Although the exact etiology of the IEH in our case remains unclear, it was likely multifactorial, related to ongoing anticoagulation, retching, and/or trauma from preceding NGT placement. The pleural involvement is extremely uncommon, and while the pathophysiology remains uncertain, it may relate to esophageal perforation with spontaneous healing.

## DISCLOSURES

Financial disclosure: None to report.

Previous presentation: This case was presented at the 2020 Virtual Annual Meeting of the American College of Gastroenterology; October 2020.

Informed consent was obtained for this case report.
